# Comparative analysis of cardiac function before LVAD implantation in patients with and without early, acute right heart failure: insights from cardiac magnetic resonance

**DOI:** 10.3389/fcvm.2025.1629252

**Published:** 2025-09-24

**Authors:** Carl-Thaddäus Braun, Hermann Körperich, Michiel Morshuis, Sabina P. W. Guenther, Lech Paluszkiewicz, Nikolai Hulde, Henrik Fox, Sebastian V. Rojas, Jan Gummert, René Schramm

**Affiliations:** ^1^ Clinic for Thoracic and Cardiovascular Surgery, Heart and Diabetes Center North Rhine Westphalia, Ruhr University Bochum, Bad Oeynhausen, Germany; ^2^ Institute for Radiology, Nuclear Medicine and Molecular Imaging, Heart and Diabetes Center North Rhine-Westphalia, Ruhr University Bochum, Bad Oeynhausen, Germany; ^3^ Institute for Anesthesiology and Pain Therapy, Heart and Diabetes Center North Rhine-Westphalia, Ruhr University Bochum, Bad Oeynhausen, Germany

**Keywords:** LVAD, CMR, right heart failure, cardiovascular imaging, heart failure

## Abstract

**Background:**

Early acute right heart failure (eaRHF) during left ventricular assist device (LVAD) implantation significantly impacts patient survival and complicates perioperative management. Although numerous clinical, echocardiographic, and hemodynamic risk factors have been identified, accurately predicting eaRHF remains challenging. Cardiac magnetic resonance (CMR) provides a precise, non-invasive evaluation of cardiac structure and function and may enhance risk stratification eaRHF. This study aims to assess the predictive value of preoperative CMR-derived parameters, comparing their utility to established echocardiographic and right heart catheterization (RHC) markers for identifying eaRHF.

**Methods:**

This retrospective analysis was conducted on 55 patients who received CMR before LVAD implantation at our center between 2018 and 2024. Of these 55 patients, 40 had image quality sufficient for offline analysis. Patients receiving a temporary right ventricular assist device (tRVAD) intraoperatively were defined as having eaRHF. Receiver Operating Characteristic (ROC) analysis was used to evaluate the predictive capability of CMR, echocardiographic, and RHC parameters.

**Results:**

Ten patients (25%) developed eaRHF. Preoperative bilirubin levels were significantly higher in the eaRHF group (1.6 mg/dl vs. 1.1 mg/dl, *p* = 0.010). Echocardiographic Tricuspid Annular Plane Systolic Excursion (TAPSE) tended to be lower in eaRHF patients (12 mm vs. 18 mm, *p* = 0.080). RHC parameters, specifically right ventricular stroke work index (RV-SWI; *p* < 0.001), cardiac output (CO; *p* = 0.003), and cardiac index (CI; *p* = 0.004), were significantly lower in eaRHF patients. CMR showed significantly higher RV end-diastolic volumes (RV-EDV, 288.4 ml vs. 216.7 ml, *p* = 0.046) and indexed RV-EDV (RV-EDVi, 135.4 ml/m^2^ vs. 104.7 ml/m^2^, *p* = 0.033) in the eaRHF group. ROC analysis identified CO (AUC = 0.90, sensitivity = 100%, specificity = 72%, *p* < 0.001), CI (AUC = 0.88, sensitivity = 83%, specificity = 83%, *p* < 0.001), and RV-SWI (AUC = 0.86, sensitivity = 83%, specificity = 86%, *p* < 0.001) as strong predictors. Moderate predictive values were observed for RV-EDVi (AUC = 0.73, *p* = 0.040) and RV global radial strain (RV-GRS; AUC = 0.70, *p* = 0.044).

**Conclusion:**

Hemodynamic parameters from RHC demonstrated the strongest predictive capability for eaRHF. However, selected CMR-derived parameters, especially indexed RV-EDV and RV GRS, offer moderate predictive value and may serve as adjunctive tools in preoperative risk stratification for LVAD candidates.

## 1 Background

Right heart failure (RHF) during left ventricular assist device (LVAD) implantation remains a prevalent, but poorly understood clinical problem that has a detrimental impact on long-term survival (1). Often, the failing right ventricle (RV) necessitates intraoperative implantation of a temporary right ventricular assist device (tRVAD), complicating patient management (2). Many echocardiographic and hemodynamic risk factors for RHF have been identified; however, predicting RHF in this heterogeneous patient population has posed a challenge to clinicians and researchers (3).

Transthoracic echocardiography (TTE) remains the most commonly used imaging modality before LVAD implantation. Therefore, most imaging risk factors, such as tricuspid annular plane systolic excursion (TAPSE), are commonly derived from echocardiographic examinations. Right heart catheterisation (RHC) is used to evaluate right heart function in patients before LVAD implantation (4). Cardiac magnetic resonance imaging (CMR) is recognized as the gold standard for precise, non-invasive analysis of cardiac structure and function (5). Utilizing this advanced imaging technique in this complex patient cohort, CMR allows for accurate measurement of cardiac functional parameters and RV strain analysis.

Given the persistent challenge of predicting eaRHF after LVAD implantation and the limited evidence on the role of CMR in this setting, our study aimed to evaluate whether preoperative CMR-derived volumetric and functional parameters could help identify patients at risk of eaRHF. By directly comparing CMR metrics with established echocardiographic and right heart catheterization (RHC) markers, we sought to determine the relative predictive value of advanced imaging vs. invasive hemodynamics and to explore whether CMR can provide incremental insights into the mechanisms underlying eaRHF.

## 2 Materials and methods

### 2.1 Study population

This single-center retrospective study included 55 patients who underwent CMR preoperatively before LVAD implantation between 2018 and 2024. CMR was performed 55 days (15–403 days) before implantation. Patients had to be older than 18 years old and not suffer from congenital cardiac abnormalities. Patients with insufficient image quality, for example, artefacts affecting strain analysis, were excluded. Baseline clinical characteristics of the included and excluded patients are presented in Supplementary Table S1. Ethical approval was obtained from the local ethics committee, and written consent was waived.

### 2.2 Right heart failure

According to the 2020 Mechanical Circulatory Support-Academic Research Consortium (MCS-ARC) guidelines, patients requiring a tRVAD implantation during the operation after LVAD implantation are defined as having early, acute right heart failure (eaRHF) (6). A CentriMag Pump (Levitronix LLC, Waltham, MA, USA) was used to assist the RV temporarily.

### 2.3 Clinical and hemodynamic data

Clinical data were collected from the patients using the electronic medical record (EMR). All patients underwent a trans-thoracic echocardiogram (TTE) before surgery. 35 patients received an RHC preoperatively, and 5 patients did not due to hemodynamic instability.

### 2.4 Cardiovascular magnetic resonance

CMR imaging was acquired using routine clinical scanners with a magnetic field strength of 1.5 or 3 Tesla. A dedicated cardiac phased-array coil was used for signal reception. All examinations were performed following the CMR imaging guidelines of the Society for Cardiovascular Resonance Imaging (7). All routine examinations include steady-state free-precession (SSFP) 4-chamber (3 slices) and short-axis (SAX) views covering the entire area of the right ventricle (RV) (12–16 slices). Each CMR examination had 25 to 40 phases per cardiac cycle. Participants were evaluated while lying in the supine position. A vector electrocardiogram was used to carry out acquisitions triggered by cardiac activity.

#### 2.4.1 Calculation and analysis of CMR parameters

Offline analysis of all images was performed using the CVI42 software package (Circle Cardiovascular Imaging Inc., Calgary, Canada, Release 5.12.1) using an external workstation by one examiner experienced in offline CMR analysis. The accuracy of the strain parameters was assessed by intraobserver variability. Volumetric values were calculated and indexed to the body surface area (BSA), which was calculated using the DuBois formula.

**Figure 1:**
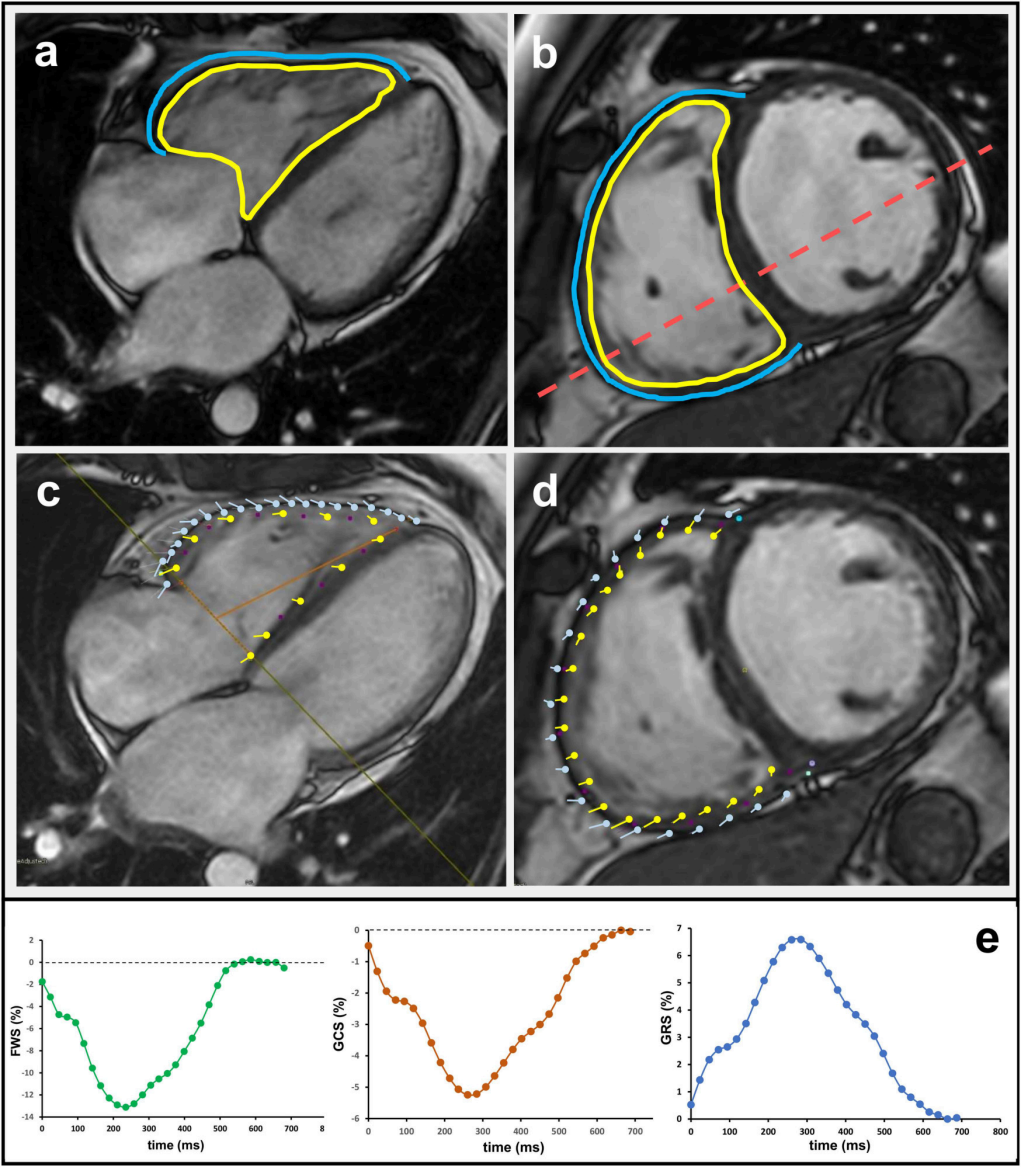
Cardiac magnetic resonance analysis of right ventricular function and strain in the 4-chamber **(a,c)** and short-axis view **(b,d)**. The yellow line marks the endocardial and the blue line the epicardial contour. Respective strain curves for right ventricular free wall strain (RV FWS), global radial strain (GRS), and global circumferential strain (GCS) are also shown.

The biplane module of the software package was utilized to calculate the volumes of the right atrium (RA). The software package automatically calculated the RA cardiac index (CI), and RA strain. The SAX cine stack was utilized to calculate the right ventricular end-diastolic (RVEDV) and right ventricular end-systolic volumes (RVESV) (8). The region of interest (ROI) was automatically calculated by the software package for the RV endocardial border. The epicardial border of the RV had to be drawn manually. If necessary, the examiner adjusted RV borders (Figure 1a). Right ventricular ejection fraction (RVEF), cardiac output (C0), and CI for the RV were automatically calculated based on the volumetric measurements (9). The examiner manually measured the end-systolic and end-diastolic phases of the RV (10). These measurements were used to calculate right ventricular end-systolic area (RVESA) and right ventricular end-diastolic area (RVEDA), as well as fractional area change (FAC). For the RV, free wall strain (FWS), global circumferential strain (GCS), and global radial strain (GRS) were calculated based on cine steady-state free-precession acquisitions. For this purpose, the 4-chamber and short-axis views were utilized (Figures 1b–e). The software settings were adjusted for optimal contour recognition and temporal smoothing, with manual correction of contours as needed to ensure accuracy in the strain measurements (11).

### 2.5 Statistics

All statistical analyses were performed using R software (R Foundation for Statistical Computing, Vienna, Austria, version 4.4.2). Due to the small sample size, data are expressed as medians with 25th and 75th percentiles. Differences between the two groups were assessed using the Mann–Whitney U test, with statistical significance set at a *p*-value <0.05. Categorical variables were compared between groups using Fisher's exact test. Receiver Operating Characteristic (ROC) curve analysis was performed to identify markers predictive of eaRHF. The area under the curve (AUC), 95% confidence intervals (using DeLong's method, with additional bootstrap resampling of 2,000 replicates), optimal cut-off values (Youden index), as well as sensitivity and specificity, were calculated using the pROC package in R. Data handling was performed using the readxl and openxlsx packages. Intraobserver reproducibility of CMR-derived strain parameters (GRS, GCS, and FWS) was assessed using intraclass correlation coefficients (ICC) and Bland–Altman analyses.

## 3 Results

### 3.1 Baseline characteristics

Of the 55 patients who were included, 40 (49.2 ± 15.2 years, 83% male) had sufficient image quality for offline strain analysis. 10 patients in the study group developed eaRHF. These patients had a higher preoperative bilirubin level [1.1 (0.6–1.4) mg/dl vs. 1.6 (1.3–1.9) mg/dl, *p* = 0.010]. Otherwise, there was no statistically significant difference between the groups. Baseline clinical characteristics are shown in Table 1.

**Table 1:** Baseline clinical characteristics of the study groups.

Variable	No RHF (Number/percentage) *N* = 30	Early, acute RHF^c^ (Number/percentage) *N* = 10	*P*-Value
BMI (kg/m^2^)^a^	25.2 (22.3–23.2)	25.1 (22.4–27.7)	0.770
BSA (m^2^)^a^	2.0 (1.8–2.2)	1.9 (1.8–2.1)	0.408
Age at Implant^a^	53.5 (47–63.8)	41.3 (34.9–46.7)	0.098
Intermacs score^b^			
1	6 (20)	2 (20)	1.000
2	9 (30)	5 (50)	0.498
3	4 (13)	3 (30)	0.377
4	11 (37)	0 (0)	0.094
Device Type^b^			
Heart Mate 3	24 (80)	6 (60)	0.776
HVAD	6 (20)	4 (40)	0.436
Etiology^b^			
ICM	9 (30)	1 (10)	0.423
DCM	21 (70)	9 (90)	0.787
Pre-OP GFR^a^ (ml/min/1.73 m^2^)	51.5 (41.3–85.5)	75 (56.8–119)	0.098
Pre-OP Bilirubin^a^ (mg/dl)	1.1 (0.6–1.4)	1.6 (1.3–1.9)	0.010
HR/min during CMR^a^	85 (70–97)	96 (77–111)	0.333

^a^ Median and 25th and 75th percentile.

^b^ Number and percentage; RHF, right heart failure; BMI, body mass index; BSA, body surface area; ICM, ischemic cardiomyopathy; DCM, dilated cardiomyopathy; HVAD, HeartWare Ventricular Assist Device; GFR, glomerular filtration rate; HR, heart rate; CMR, cardiac magnetic resonance.

^c^ According to the 2020 MCS-ARC definition (5).

### 3.2 Echocardiographic and right heart catheterisation data

The echocardiographic examination was performed 7.5 (3–12) days before LVAD implantation. TAPSE tended to be lower in patients with eaRHF [12 (11.8–14.3) mm vs. 18 (12–19) mm, *p* = 0.080]. Otherwise, there were no differences between the groups (Table 2). RHC was performed 6 (2–11) days before implantation. Patients with eaRHF had a lower RV-SWI [13.9 (11.2–15.1) g·m/m^2^ vs. 22.9 (20–28.8) g·m/m^2^, *p* < 0.001]. Furthermore, these patients had lower CO [2.3 (2.2–3) L/min vs. 4.1 (3–4.9) L/min, *p* = 0.003] and CI [1.2 (1–1.5) L/min/m^2^ vs. 1.9 (1.8–2.5) L/min/m^2^, *p* = 0.004] as well as a tendency toward lower PAPi. The results of the RHC are shown in Table 3.

**Table 2:** Echocardiographic parameters of the study group.

Variable	No RHF *N* = 30	RHF *N* = 10	*p*-Value
TAPSE (mm)	18 (12–19)	12 (11.8–14.3)	0.082
TI > 1	13 (43)	5 (50)	0.709
LVEF (%)	24 (19–30)	20 (18–25)	0.229
LVEDD (mm)	71 (63–72)	68 (60–77)	0.853
RVEDD (mm)	45 (41–50)	45 (41–51)	0.804
RVEDD:LVEDD	0.68 (0.61–0.76)	0.71 (0.6–0.75)	0.825

TAPSE, tricuspid annular plane systolic excursion; TI, tricuspid valve insufficiency; LVEF, left ventricular ejection fraction; LVEDD, left ventricular end-diastolic diameter; RVEDD, right ventricular end-diastolic diameter.

**Table 3:** Right heart catheter data of the study group. In 5 patients, right heart catheterization was not performed due to hemodynamic instability.

Variable	No RHF *N* = 29	RHF *N* = 6	*p*-Value
Heart Rate (beats/min)	87 (75–100)	103 (90–110)	0.197
RV-SWI (g m^−2^ beat^−1^)	6.1 (4.8–8.5)	2.1 (0.9 8.5)	0.004
PCWP (mmHg)	21 (17–34)	22 (19.5–26)	0.884
CVP (mmHg)	12 (7–16)	18 (11–23)	0.189
CO (L/min)	4.1 (3–4.9)	2.3 (2.2–3)	0.003
CI (L/min/m^2^)	1.9 (1.8–2.5)	1.2 (1 -1.5)	0.004
PAPm (mmHg)	38 (24 -45)	27 (25–33)	0.277
PAPs (mmHg)	49 (31–61)	32 (29–42)	0.298
PAPd (mmHg)	26 (20–36)	24 (22–27)	0.572
PAPi	1.74 (1.2–1.68)	0.91 (0.3–1.58)	0.082
CVP/PCWP ratio.	0.49 (0.34–0.69)	0.68 (0.42–0.91)	0.183

RV-SWO, right ventricular stroke work index; PCWP, pulmonary capillary wedge pressure; CVP, central venous pressure; CO, cardiac output; CI, cardiac index; PAPm, mean pulmonary artery pressure; PAPs, systolic pulmonary artery pressure; PAPd, diastolic pulmonary artery pressure; PAPi, pulmonary artery pressure index.

### 3.3 Assessment of right atrial and right ventricular morphology and function using CMR

RA and RV morphology and function were assessed using CMR. When both groups were compared, there were no differences in RA strain [RHF: 7.9 (3.6–22.7) vs. no RHF: 6.7 (5.3–15.2), *p* = 1.000]. No differences were seen in the other RA parameters measured with CMR (Table 4). When looking at RV function, patients with eaRHF tended to have a higher ESV [165.1(93.5–231.8) ml vs. 226.5 (161.8–260.2) ml, *p* = 0.061] and ESVi [85.9 (51.4–117.7) ml/m^2^ vs. 123.6 (87.7–134.8) ml/m^2^, *p* = 0.080], but there was no statistically significant difference. The EDV [216.7 (143.4–255.1) ml vs. 288.4 (192.3–307.7) ml, *p* = 0.046] and EDVi [104.7 (76.9–122.6) ml/m^2^ vs. 135.4 (114–163.9) ml/m^2^, *p* = 0.003], however, were statistically higher in patients with eaRHF compared to patients without eaRHF. Furthermore, RV-GRS tended to be lower in patients with eaRHF [9.2 (5.4–14.8) % vs. 4.4 (3.7–7.4) %, *p* = 0.065]. Table 5 shows the results of RV parameters measured with CMR.

**Table 4:** Right atrial functional parameters assessed using cardiac magnetic resonance.

Variable	No RHF *N* = 30	RHF *N* = 10	*p*-Value
RA ESV (ml)	75.8 (43.6–128.2)	101 (62.2–137.3)	0.453
RA-ESVi (ml/m^2^)	41.8 (23.4–61.9)	50 (31.4–63.7)	0.563
RA EDV (ml)	93.1 (68.7–137.9)	118.5 (86.5–143.9)	0.453
RA EDVi (ml/m^2^)	48.4 (32–74.2)	57.5 (45.9–69.8)	0.453
RA EF (%)	18.1 (6.3–28.1)	16 (4.2–27.5)	0.803
RA strain (%)	7.9 (3.6–22.7)	6.7 (5.3–15.2)	1.000

RHF, right heart failure; RA, right atrium; ESV, end-systolic volume; ESVi, indexed end-systolic volume; EDV, end-diastolic volume; SV, stroke volume; SVi, stroke volume index; EDVi, indexed end-diastolic volume; CI, cardiac index; EF, ejection fraction.

**Table 5:** Right ventricular parameters measured with cardiac magnetic resonance.

Variable	No RHF *N* = 30	RHF *N* = 10	*p*-Value
RV-EDV (ml)	216.7 (143.4–255.1)	288.4 (192.3–307.7)	0.046
RV-EDVi (ml/m^2^)	104.7 (76.9–122.6)	135.4 (114–163.9)	0.033
RV-ESV (ml)	165.1 (93.5–231.8)	226.5 (161.8–260.2)	0.067
RV-ESVi (ml/m^2^)	85.9 (51.4–117.7)	123.6 (87.7–134.8)	0.080
RV-SV (ml)	33.8 (17.2–66.6)	23.4 (15.3–34.3)	0.310
RV-SVi (ml/m^2^)	17.3 (10–31.8)	11.2 (8.7–18.8)	0.348
RV-EF (%)	20.7 (9.5–33.4)	13.2 (6.7–19.3)	0.206
RV-CO (L/min)	3.3 (1.6–5)	2.5 (1.2–4.8)	0.851
RV-CI (L/min/m^2^)	1.6 (0.8–2.5)	1.4 (0.6–2.2)	0.766
TAPSE (mm)	9.7 (7.6–13.1)	9.5 (6.2–13.1)	0.925
RV-EDA (cm^2^)	33.8 (26.7–38.8)	35.4 (30–41.7)	0.463
RV-ESA (cm^2^)	28.1 (14.6–31.2)	27.9 (25.8–32.2)	0.522
RV-FAC (%)	22.6 (14.3–40.8)	15.7 (13.3–25)	0.261
RV-FWS (%)	−10.9 (−16.1 to (−8.1))	−7.5 (−10.4 to (−6.7))	0.130
RV-GCS (%)	−5.5 (−8.4 to (−3.6))	−3.3 (−5.1 to (−2.2))	0.118
RV-GRS (%)	9.2 (5.4–14.8)	4.4 (3.7–7.4)	0.065

RHF, right heart failure; RV, right ventricle; EDV, end-diastolic volume; EDVi, indexed end-diastolic volume; ESV, end-systolic volume; ESVi, indexed end-systolic volume; SV, stroke volume; SVi, indexed stroke volume; EF, ejection fraction; CO, cardiac output; CI, cardiac index; TAPSE, tricuspid annular plane systolic excursion; EDA, end-diastolic area; ESA, end-systolic area; FAC, fractional area change; FWS, free wall strain; GCS, global circumferential strain; GRS, global radial strain.

### 3.4 Intraobserver variability of CMR-derived RV strain parameters

To assess intraobserver variability of RV GRS, GCS, and FWS, ICC and Bland–Altman analyses were performed. The reproducibility was excellent for all parameters with ICC (2,1) values of 0.98 (95% CI: 0.94–0.99) for GRS, 0.94 (95% CI: 0.86–0.98) for GCS, and 0.92 (95% CI: 0.81–0.97) for FWS. Bland–Altman analyses revealed small mean differences between the repeated measurements (GRS −0.18 ± 1.95%; GCS +0.27 ± 1.61%; FWS −0.38 ± 2.69%), with no systematic bias observed (all paired comparisons: *p* > 0.45).

### 3.5 Receiver-operating-curve analysis

**Figure 2:**
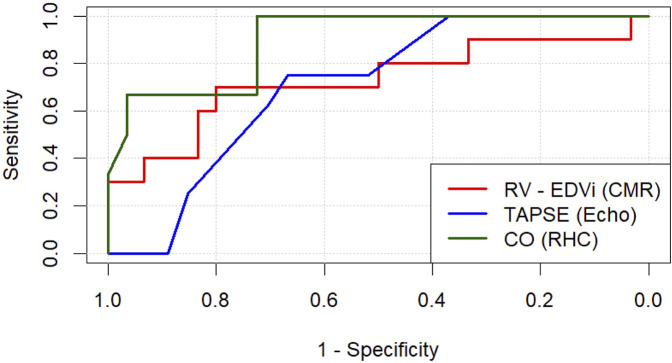
Receiver operating characteristic (ROC) analysis is shown for the three most predictive parameters for each respective modality studied: cardiac magnetic resonance (CMR), echocardiography (echo) and right heart catheter (RHC). RV-EDVi, right ventricular end-diastolic volume index; TAPSE, tricuspid anular plane systolic excursion.

ROC curve analysis was conducted to evaluate the predictive ability of CMR, echocardiographic and RHC parameters (Table 6). Among CMR parameters, RV EDVi showed moderate predictive capability (AUC = 0.73, *p* = 0.040), achieving a sensitivity of 70% and a specificity of 80% at a threshold of 129.7 ml/m^2^. RV GRS also demonstrated moderate predictive performance (AUC = 0.70, sensitivity = 70%, specificity = 80%, threshold = 5.25%, *p* = 0.044). Echocardiographic-derived TAPSE exhibited moderate predictive value (AUC = 0.71, sensitivity = 75%, specificity = 67%, threshold = 14.0 mm, *p* = 0.022). Parameters derived from RHC had the strongest predictive performance, with CO yielding an excellent prediction (AUC = 0.90, sensitivity = 100%, specificity = 72%, threshold = 3.31 L/min, *p* < 0.001). CI also demonstrated high predictive accuracy (AUC = 0.88, sensitivity = 83%, specificity = 83%, threshold = 1.55 L/min/m^2^, *p* < 0.001). Additionally, RV SWI demonstrated substantial predictive strength (AUC = 0.86, sensitivity = 83%, specificity = 86%, threshold = 3.43 g·m/m^2^, *p* < 0.001). Other CMR-derived parameters exhibited trends toward predictive capability but did not reach statistical significance. ROC curves representing the most predictive parameters are presented in Figure 2.

**Table 6:** Receiver-Operating-Curve analysis of cardiac magnetic resonance, echocardiographic and right heart catheter parameter and their ability to predict early-acute right heart failure preoperatively.

Marker	AUC	Lower CI	Upper CI	Sensitivity	Specificity	Threshold	*P*-Value
*CMR*							
RV EDV (ml)	0.71	0.48	0.95	0.60	0.97	284.6	0.073
EV ESV (ml)	0.70	0.49	0.90	0.70	0.67	190.5	0.056
RV EDVi (ml/m^2^)	0.73	0.51	0.94	0.70	0.8	129.7	0.040
RV ESVi (ml/m^2^)	0.69	0.50	0.88	0.60	0.76	119.8	0.054
RV FWS (%)	0.66	0.44	0.89	0.60	0.80	−7.65	0.152
RV GCS (%)	0.67	0.47	0.86	0.80	0.60	−5.35	0.092
RV GRS (%)	0.70	0.51	0.89	0.70	0.80	5.25	0.044
Echo							
TAPSE	0.71	0.53	0.88	0.75	0.67	14.00	0.022
RHC							
RV SWI	0.86	0.70	1.00	0.83	0.86	3.43	<0.001
CO	0.90	0.77	1.00	1.00	0.72	3.31	<0.001
CI	0.88	0.73	1.00	0.83	0.83	1.55	<0.001
PAPi	0.73	0.50	0.97	0.67	0.75	1.24	0.053

AUC, area under the curve; CI, confidence interval; CMR, cardiac magnetic resonance; RV EDV, right ventricular end-diastolic volume; RV ESV, right ventricular end-systolic volume; RV EDVi, right ventricular end-diastolic volume index; RV ESVi, right ventricular end-systolic volume index; RV FWS right ventricular free wall strain; RV GCS, right ventricular global circumferential strain; RV GRS, right ventricular global radial strain; TAPSE, tricuspid annular plane systolic excursion; RHC, right heart catheter; RV SWI, right ventricular stroke work index; CO, cardiac output; CI, cardiac index; PAPi, pulmonary artery pressure index.

## 4 Discussion

EaRHF remains a challenging clinical problem, with a significant morbidity and mortality post-LVAD implantation (2). Many patients with end-stage heart failure receive an implantable cardioverter defibrillator, which makes it more challenging to undergo CMR (12). Therefore, previous studies have traditionally mainly focused on TTE and RHC-derived risk markers, which have been used to postulate several mechanisms for RHF (13). Therefore, this is one of the few studies focusing on CMR-derived RV volumetric and functional parameters before LVAD implantation.

Contrary to prior TTE and RHC studies that identified RA strain and elevated CVP as predictors of RHF, we did not observe significant differences in RA functional parameters between groups (14, 15). Although the median CVP was higher in the eaRHF group, there was no statistically significant difference. The differences in RA strain could be attributed to the CMR biplane tool, which may have a lower temporal resolution than speckle-tracking echocardiography, potentially limiting its sensitivity to subtle RA strain abnormalities. The failure to show differences between the two groups in this study cohort may be due to RHF being primarily driven by inadequate RV adaptation, notably due to reduced RV contractility and diastolic dysfunction, rather than isolated atrial performance. In our cohort, CMR-derived RA measures may have lacked sensitivity to subtle atrial impairment that occurs in the context of evolving RV diastolic uncoupling (16).

Several mechanisms have been postulated to cause eaRHF. One of these mechanisms is that RVs with preserved diastolic compliance may fail to provide the needed SV, possibly leading to RHF (16). Furthermore, there is a disruption in the normal longitudinal contraction of the RV, leading to a gain in transverse shortening (17). In our study group, there was no statistically significant difference in RV SV and RV SVi between the study groups. RV EDV and RV EDVi, however, differed significantly between the groups. Timmons et al. demonstrated in 15 patients that RV EDVi was able to predict mortality after LVAD implantation, whereas RVEF failed to do so (18). In a previous study by Nuqali et al., CMR-derived RVEF did not predict post-LVAD RHF (19). In this patient cohort, CMR-derived RVEF also failed to predict eaRHF. It is important to note that RVEF was significantly reduced in both groups, with a median RVEF of 13.2% in the eaRHF group. This reinforces the hypothesis that traditional metrics might inadequately assess the complex interplay between RV preload, afterload, and intrinsic myocardial function under conditions of LVAD-induced hemodynamic shifts.

Studies focusing on TTE-derived RV strain have shown that FWS can predict RHF, but RV GLS fails to do so. Furthermore, cut-off values vary widely from −5 to −15%, precluding clinical implementation (20, 21). Another factor contributing to RV function is the interventricular septum (IVS). As the LVAD increases RV preload and decreases LV end-diastolic pressure, a leftward septum shift of the IVS is observed (22). Using CMR, longitudinal, radial, and contractile functions can be assessed. Measuring GCS and GRS preoperatively using CMR could give greater insights into the RV's ability to adapt to these intraoperative changes in contractile function. In this patient cohort, GRS was a moderate predictor of eaRHF, with RV FWS failing to predict RHF.

RHC data demonstrated superior predictive capability compared to CMR-derived RV parameters in our cohort. Several publications have shown the predictive ability of RVSWI and PAPi (23, 24). RHC data in this study group were captured closer to the implantation date than CMR data. Therefore, preoperative hemodynamic deterioration was probably more accurately captured with RHC than with CMR. In contrast, CMR-derived parameters, although precise in anatomical and volumetric assessment, may not fully capture the acute interplay between preload, afterload, and contractile reserve. The superiority of RHC in our cohort may reflect both the temporal proximity of assessment and the ability of invasive hemodynamics to capture the pathophysiologic processes most relevant to the immediate perioperative period.

### 4.1 Limitations

This study's limitations warrant consideration. Primarily, the retrospective design and small sample size limit generalizability and introduce potential selection bias. Pre-LVAD-implant CMR examinations in advanced heart failure patients are rare, as many patients have devices that preclude MRI. Additionally, there was significant variability in the time interval between CMR and LVAD implantation, with some patients undergoing CMR more than a year prior to surgery. Image quality issues also led to the exclusion of several patients, further introducing potential bias. Only TAPSE was included from echocardiographic measurements. Other routinely available echocardiographic parameters of RV function (e.g., RV S′, RV FAC) were not measured in all patients and could not be incorporated into the analysis. Importantly, the operational definition of early acute RHF as intraoperative temporary RVAD implantation may only identify the most severe cases. As a result, this may underestimate the overall incidence of RHF and restrict generalizability to broader RHF phenotypes. Finally, while understandable given the limited sample size, the absence of multivariable modelling restricts assessment of whether CMR parameters independently predict RHF.

## 5 Conclusion

In conclusion, our study suggests that eaRHF after LVAD implantation is driven more by RV maladaptation than by atrial dysfunction. While RHC remains superior due to its ability to capture real-time hemodynamics, CMR-derived measures such as RV EDVi and GRS showed moderate predictive value and may complement invasive assessment. Larger prospective studies are needed to confirm these findings and refine risk-stratification strategies.

## Data Availability

The raw data supporting the conclusions of this article will be made available by the authors, without undue reservation.
