# Supplementary material for: Comparative analysis of cardiac function before LVAD implantation in patients with and without early, acute right heart failure: insights from cardiac magnetic resonance
Source: Front Cardiovasc Med. 2025 Sep 24;12:1629252. doi: 10.3389/fcvm.2025.1629252 (PMC12504228; doi:10.3389/fcvm.2025.1629252)
Supplement: Supplementary file 1 [file Table1.docx]

Supplementary Table 1

Baseline clinical characteristics of the included and excluded patients in the study group.

|  | Study group  (Number/ Percentage)  N=40 | Excluded Patients  (Number/percentage)  N=15 | P-Value |
| --- | --- | --- | --- |
| BMI (kg/m^2^)^1^ | 25.2 (22.2 – 28.1) | 23.8 (20.4 – 25.8) | 0.180 |
| BSA (m^2^)^1^ | 2.0 (1.8 – 2.2) | 2.0 (1.8 – 2.1) | 0.345 |
| Age at Implant^1^ | 50 (40.5 – 63.3) | 59.5 (30.9 – 63.7) | 0.895 |
| Intermacs score^2^ |  |  |  |
| 1 | 8 (20) | 4 (27) | 0.729 |
| 2 | 14 (35) | 1 (7) | 0.163 |
| 3 | 7 (18) | 2 (13) | 1.000 |
| 4 | 11 (28) | 8 (53) | 0.259 |
| Device Type^2^ |  |  |  |
| Heart Mate 3 | 30 (75) | 9 (60) | 0.811 |
| HVAD | 10 (25) | 5 (33) | 0.749 |
| Berlin-Heart | 0(0) | 1 (7) | 0.286 |
| Etiology^2^ |  |  |  |
| ICM | 10 (25) | 4 (27) | 1.000 |
| DCM | 30 (75) | 8 (53) | 0.626 |
| Congenital | 0 (0) | 3 (20)* | 0.026 |
| Pre-OP GFR^1^ (mL/min/1.73m^2^) | 59 (42 – 90.5) | 53 (37 – 73) | 0.269 |
| Pre-OP Bilirubin^1^ (mg/dl) | 1.2 (0.8 – 1.6) | 1.2 (0.8 – 2-3) | 0.643 |

^1^Median and 25^th^ and 75^th^ percentile, ^2^Number and percentage; RHF – right heart failure, BMI – body mass index, BSA – body surface area, ICM – ischemic cardiomyopathy, DCM – dilated cardiomyopathy, HVAD – HeartWare Ventricular Assist Device, GFR – glomerular filtration rate,

*2 Patients had corrected congenital transposition of the great arteries, and one patient had Ebstein’s anomaly.
